# Effectiveness of Clomiphene Citrate for Improving Sperm Concentration: A Literature Review and Meta-Analysis

**DOI:** 10.7759/cureus.25093

**Published:** 2022-05-18

**Authors:** Dragos Puia, Catalin Pricop

**Affiliations:** 1 Urology, University for Medicine and Pharmacy "Grigore T. Popa", Iasi, ROU

**Keywords:** srems, meta-analysis, male infertility, sperm count, clomiphene

## Abstract

Demographic data regarding male infertility suggest an increase in prevalence. This is an entity with multifactorial etiology, hormonal causes are often encountered. Although treatment with clomiphene was advocated to stimulate gametogenesis, it is still used off-label. We aimed to evaluate data from literature related to the effect of clomiphene as a single therapy, on the improvement of sperm count in infertile patients. Out of the 4,017 results of the search, only eight articles have been selected. The selected studies have been published between 1983 and 2020, and have included a total of 616 patients. From data reported, the treatment with clomiphene lead to a significant improvement of sperm concentration compared with placebo or with the level before starting the therapy (p<0.00001). Out of the 616 patients, in 369 (59.90%) cases improved sperm concentration was reported. In our meta-analysis, the selected studies had a high heterogeneity (I^2^^ ^= 97%). Nevertheless, clomiphene is not an ideal treatment, paroxysmal effects have been reported. Our findings encourage the use of clomiphene on male infertility, although the potential side effects should be clearly explained to patients.

## Introduction and background

Current epidemiological data suggest an increase in the prevalence of male infertility. According to Agarwal et al., the prevalence of infertile men ranges from 2.5% to 12% depending on the geographical area [1]. There are many factors involved in male infertility. According to Machen et al., they can be classified as pre-testicular, testicular and post testicular factors. In the first category, hormones play an important role [2]. Besides elevated prolactin, hypogonadotropic hypogonadism is an endogenous cause that affects male fertility through a complex mechanism. A low luteinizing hormone (LH) leads to a low testosterone influence negatively the sperm production, while a low follicle-stimulating hormone (FSH) affects Sertoli/germ cell function. From this point of view, it seems rational to use various hormone-acting drugs for the treatment of male infertility.

The projected mechanism of clomiphene, selective estrogen receptor modulators (SERMs), relies on the activity of those compounds to dam steroid receptors at the extent of the neural structure, which ends up in stimulation of GnRH secretion resulting in a rise in pituitary gonadotrophic hormone unharness. The latter impact, by stimulating gametogenesis, represents the rational basis for SERM administration to patients with reduced sperm cell count. Although some positive results have been reported for the utilization of SERMs in men with the upset physiological condition are rumored, no conclusive recommendations are often drawn because of the poor quality of the studies. What is more, complications from the utilization of SERMs were under-reported. According to the 2022 European Urology Guideline, the recommendations for the use of SERMs in men with idiopathic infertility are weak [3]. We aimed to evaluate data from literature related to the effect of clomiphene as a single therapy, on the improvement of sperm count in infertile patients.

## Review

We performed this meta-analysis and literature review using Preferred Reporting Items for Systematic Reviews and Meta-Analyses (PRISMA) reporting guidelines. A systematic search of Medline and Embase databases was performed, using the following words: “clomiphene” (MeSH Terms) AND (“sperm count” [All Fields] OR (“oligospermia” [All Fields] OR “male infertility” [All Fields]). We did not include non-English language articles and those in whom the full text was not available. The main search, as well as screening for eligibility of titles, abstracts, and full-text articles, was completed independently by two authors, and any discrepancies were solved by consensus. Articles that included patients with diseases that are known to be responsible for male infertility (e.g., Klinefelter syndrome, Kartagener's syndrome, history of chemotherapy) were excluded.

We have assessed heterogeneity using I^2^ statistics. To calculate the individual odds ratio (OR) and individual and pooled mean differences with corresponding 95% CI we used Review Manager (RevMan), Version 5.4, The Cochrane Collaboration, 2020. To calculate the mean difference, we compared the outcomes of sperm count after treatment with clomiphene as a single therapy, either placebo-controlled or not. We applied standard a random effect model.

Results

**Figure 1:**
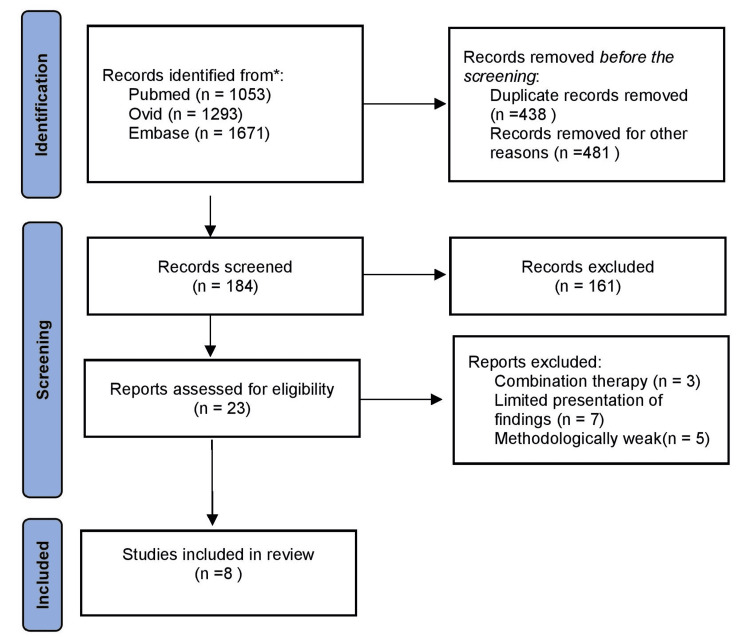
PRISMA flowchart of the study selection

Out of the 4,017 results of the search, only 8 articles have been selected, the flowchart of selection is shown in Figure 1. The selected studies have been published between 1983 and 2020, and have included a total of 616 patients. The PRISMA flowchart of the study selection is shown in Figure 1. The characteristics of included studies are present in Table 1.

**Table 1:** Characteristics of included studies

Author	Year of publication	No. of patients	Dosage	Duration of treatment	Placebo-controlled
Wang et al. [4]	1983	18	25 mg/day	6 months	yes
Mičič et al. [5]	1985	101	50 mg/day	6-9 months	yes
Sokol et al. [6]	1988	20	25 mg/day	12 months	yes
Hommonai et al. [7]	1988	44	25 mg/day	4-5 months	no
Kim et al. [8]	2016	171	12.5/25 mg/day	17 weeks	yes
Surbone et al. [9]	2019	18	50 mg/48h	3 months	no
Sharma et al. [10]	2019	44	25 mg/day	2.8 months	no
Mandal et al. [11]	2020	200	25 mg/day	3 months	yes

**Figure 2:**
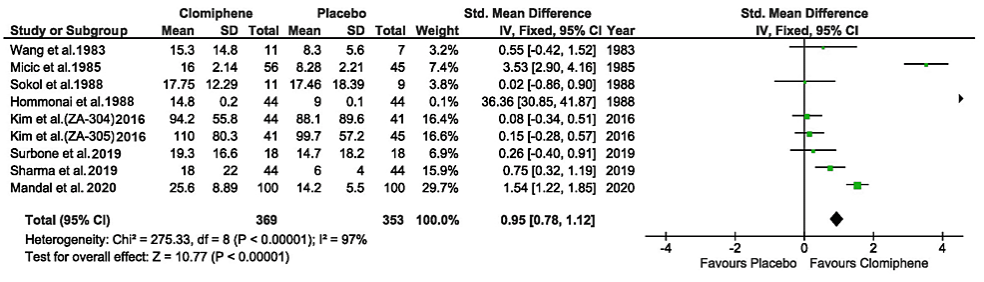
Forest plot of the treatment outcome Wang et al.* *[4], Mičič et al. [5],  Sokol et al. [6], Hommonai et al. [7], Kim et al. [8], Surbone et al.[9], Sharma et al. [10], Mandal et al. [11]

**Figure 3:**
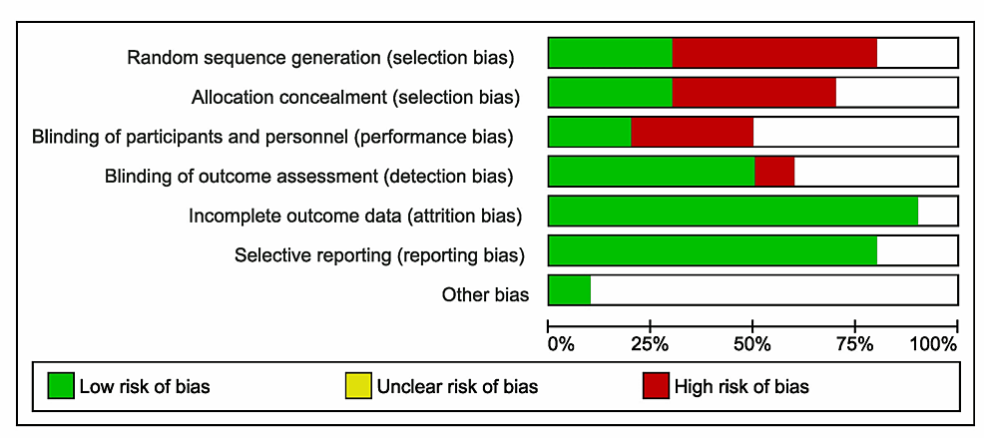
Risk of bias graph

Three of the included studies did not have a placebo arm, the data representing the before and after results. Also one of them included an arm with patients whose spermogram was normal, their data was not included in the forest plot [10]. One study reported two parallel dosage regimens (12.5 mg/day and 25 mg/day respectively), the results have been included separately in the forest plot [8]. From data reported, the treatment with clomiphene lead to a significant improvement of sperm concentration compared with placebo or with the level before starting the therapy (p<0.00001). Out of the 616 patients, in 369 (59.90%) cases improved sperm concentration was reported. In our meta-analysis, the selected studies had a high heterogeneity (I^2^ = 97%), as shown in the forest plot (Figure 2). Figure 3 represents the risk of bias graph.

Discussions

According to Papanikolaou et al., 40% of patients investigated for infertility are diagnosed with hypogonadism. It influences infertility depending on when it is installed, pre or postpubertal [12]. In addition, in many men, testosterone starts to drop after the age of 30. According to Raheem et al., to confirm the diagnosis of hypogonadism, it is not enough for the patient to have low levels of total serum testosterone but also clinical signs [13]. Therefore, although it would seem logical as the main treatment be testosterone replacement therapy. Paradoxically, according to Stewart et al., this leads to inhibition of the hypothalamic-pituitary-gonadal axis resulting in inhibition of spermatogenesis [14]. Also, in a multicenter study by the World Health Organization, testosterone treatment had contraceptive effects, 65% of men had become azoospermic after six months [15]. To avoid this, some alternative therapies are available, which include gonadotropins, aromatase inhibitors and last but not least SERMs.

Gonadotropins are used to increase endogenous testosterone production. One of the first studies on this subject have been published by Jacobson et al. in 1979 [16]. It is administered subcutaneously in doses of 1,500-3,000 IU 1-3 times weekly and, according to La Vignera et al., it is superior to testosterone replacement therapy regarding sperm concentration and the number of spermatozoa with progressive motility while stimulating the testosterone production [17]. Another off-label treatment for hypogonadism is aromatase inhibitors. In a group of 27 males treated with letrozole 2.5 mg/day for more than six months, Saylam et al. reported a significant total testosterone increase with a decrease of estradiol while the sperm count and ejaculate volume improved significantly [18]. Helo et al. compared 26 males with the effect of the aromatase inhibitors and SERMs. After 12 weeks of treatment, clomiphene has been superior for increasing the testosterone levels, whereas anastrozole significantly increased the testosterone/estradiol ratio [19].

Although it is still prescribed off-label, according to Chu et al., clomiphene citrate is one of the most popular drugs for male infertility due to its low cost and oral administration [20]. Some authors have suggested that it would act on Leydig cells by stimulating testosterone production and interfering with the action of xenoestrogens. Clomiphene seems to be the first choice for the empiric treatment of male infertility. In a survey by Ko et al., that included 191 urologists from the United States out of which only 29 (17.7%) were reproductive urologists, 94.8% of non-reproductive urologists and 100% of reproductive urologists considered clomiphene citrate as the best medication for nonobese patients with idiopathic oligospermia. In obese patients (67.9%), 62.1% of responders consider it the most suitable treatment. According to the authors for the urologist in the US, the main reason to initiate empiric therapy is low sperm count and secondly the FSH levels [21].

Being an off-label treatment there is also no consensus regarding the optimal dosage, although from the data published so far it seems that a maximum dose of 25 mg/day would be most effective. Hommonai et al. compared a dose of 25 mg daily with 25 mg on alternate days, with patients in the second group showing a superior improvement, while Kim et al. reported better effectiveness regarding the sperm count in patients who had received 25 mg/day compared with those treated with 12.5 mg/day [7,8]. High doses of clomiphene seem to have the same paradoxical effect. In a group of 53 hypofertile males who received 100 mg of clomiphene on alternate days for 3-15 months, Ross et al. reported a decrease in sperm concentration in 16.98% of patients with an average decrease of 47.9% [22].

The effect of clomiphene on sperm concentration was also evaluated by some authors in combination with antioxidants. In the group of 60 subfertile males, Ghanem et al. compared the impact of therapy with a combination of clomiphene and vitamin E to a placebo. Patients were split into two groups, one receiving a combination of clomiphene citrate (25 mg/day) and vitamin E (400 mg/day) and the other receiving a placebo (n=30). The treatment was continued for six months. When compared to the placebo group, the treated group had a substantial increase in sperm concentration and forward progression motility [23]. Other authors have reported different results, Bozhedomov et al., in a cohort of 173 infertile males, noticed that by adding L-carnitine fumarate (1 g), acetyl-L-carnitine (0.5 g) twice daily to the clomiphene therapy did not improve the morphology, progressive sperm motility and pregnancy rates compared to monotherapy after six months [24].

However, not all authors agree on the potential benefits of clomiphene usage. Paqualotto et al. reported a series of three patients with severe oligozoospermia who become azoospermic after the use of clomiphene. Three months after discontinuing the treatment they had a mean sperm concentration of 2.5 ± 1.1×10^6^/mL [25]. Although it is used off-label for the treatment of subfertile males for more than six decades, paradoxical effects have been reported in very few studies. According to Gundewar et al., the possible explanations are attributed to technical errors, sperm analysis is a highly variable biological measure. In almost 10% of cases with abnormal semen at first evaluation, a second one, without any treatment can reveal a normal sperm count. Furthermore, clomiphene could act directly on testicular histology and not least by increasing estrogen concentration [26].

Given that it is a drug designed for women but used off-label for men, one of the concerns is systemic side effects. According to Earl et al., the most reported adverse effect is venous thromboembolism, although the incidence is very low. Other side effects recorded in phase II trials have been nausea (3.3%), diarrhea (2.1%), nasopharyngitis (1.9%), or dizziness (1%). Furthermore, according to the authors, the dose (12.5 mg vs. 25 mg) was not an element that influenced the rate of adverse events [27]. Also, in a group of 400 hypogonadal males who received treatment with clomiphene citrate for a mean period of 20.48 months, Krzastek et al. reported only minor side effects like mood changes (4.16%), blurred vision (2.5%) and breast tenderness (1.6%) [28]. Also, from our own experience, after starting the treatment with a dose of 25 mg daily for a period of up to three months in over 50 patients, none of the patients reported significant side effects.

Although first data regarding the empiric use of clomiphene have been published in the mid-1960s, to this day no consensus has been reached on its efficacy because there are few placebo-controlled studies and existing ones include a small number of patients. Furthermore, in some patients, clomiphene treatment has been associated with side effects. According to Kavoussi et al., hyperestrogenemia has been encountered in many patients and it had to co-administrate aromatase inhibitors. Other reported side effects have been fatigue, gynecomastia and weight gain [29]. However, according to Sharma et al., safe for long‐term treatment, clomiphene is safer and more cost-effective when compared to testosterone replacement therapy [10].

## Conclusions

Clomiphene remains a poorly investigated drug for the treatment of some forms of male infertility. The majority of research show that clomiphene is typically considered safe to give to hypofertile patients. Clomiphene therapy and its influence on sperm parameters have yielded conflicting results across the literature. The different results of the studies are also influenced by different inclusion and exclusion criteria as well as by the different dosage used for treatment. This could be because clomiphene is still an off-label medication used to treat infertility and hypogonadism. Also, before initiating treatment with clomiphene, patients should be thoroughly investigated for the etiology of infertility, as this may influence the response to treatment. Our findings encourage the use of clomiphene on male infertility, although the potential side effects should be clearly explained to patients. Further randomized control trials, preferably placebo-controlled, are necessary to determine the efficacy, safety and optimal dose of this type of treatment.
